# Sirt3 regulates NLRP3 and participates in the effects of plantainoside D on acute lung injury sepsis

**DOI:** 10.18632/aging.204628

**Published:** 2023-04-05

**Authors:** Jing Wang, Wanrong Li, Fang Zhao, Qianqian Han, Lingling Shan, Yumei Qian

**Affiliations:** 1 School of Biology and Food Engineering, Institute of Pharmaceutical Pharmacology Research Center, Suzhou University, Suzhou, Anhui, China

**Keywords:** sirt3, NLRP3, sepsis, ALI

## Abstract

Sepsis, a common critical disease, has high morbidity and mortality. Acute lung injury (ALI) is one of the important complications of sepsis, its effective treatment measures remain scarce. The purpose of the present study was to search for the biomarker and effective treatment measures. Lipopolysaccharide (LPS) was used to establish sepsis induced ALI model *in vivo* and *in vitro*. Proteomics, immunoprecipitation, molecular docking techniques, and Sirt3 knockout (KO) mice and silence MLE-12 cells were used to search for biomarker and treatment measures for sepsis ALI. 38 differentially expressed proteins were found in the lung tissues of sepsis ALI mice, among which Sirt3 changed most. Further study found that Sirt3 could inhibit NLRP3 activation. Sirt3 KO or silence significantly aggravated sepsis induced ALI and MLE-12 cell injury. Plantainoside D (PD), an effective component of *Plantago asiatica L.*, significantly improved sepsis induced ALI by regulation of Sirt3/NLRP3 pathway. In conclusion, Sirt3 may be the important molecular targets for sepsis ALI. PD could protect sepsis ALI via Sirt3/NLRP3 signal pathway. The findings provide a new treatment target for sepsis ALI and a potential treatment measure.

## INTRODUCTION

Sepsis is a systemic inflammatory syndrome caused by infection, in which inflammatory cells over secrete inflammatory mediators and cytokines, leading to immune dysfunction and impairment of tissue and organ functions. The lung is one of the first target organs damaged in sepsis patients, which usually leads to ALI. The incidence rate and mortality of sepsis ALI are both high, and it is a hot and difficult research topic [1–3]. Sepsis is a runaway inflammatory reaction. A large number of inflammatory cells infiltrate the lung tissue. On the one hand, it starts from TNF-α cascade reaction led to a large number of neutrophils were gathering in the lungs and prompt the release of other inflammatory factors, causes lung injury. On the other hand, neutrophils release elastase, promoting a large number of neutrophils into the lung tissue, causing pulmonary edema, protein leakage, and exacerbating lung injury [4]. LPS directly stimulates neutrophils to release inflammatory mediators and elastase, which is an important pathogenic molecule leading to sepsis ALI. The key to treatment is to quickly and effectively control or reduce the excessive inflammatory response of the lung [5]. At present, glucocorticoids are the most frequently used in the treatment of sepsis acute lung injury. Although glucocorticoids have obvious effects, they have significant side effects. Traditional Chinese Medicine (TCM), which has obvious clinical effects and simple methods of operation, has been put into clinical practice in nearly 200 countries and regions around the world [6]. Plantainoside D (PD), an effective component of *Plantago asiatica L.* has the effects of clearing heat, detoxifying, detumescence and pain relief, and has certain clinical significance in the treatment of osteoarthritis [7, 8]. However, there are few reports about the effect of PD on sepsis ALI.

## RESULTS

### Proteomic analysis results

**Figure 1:**
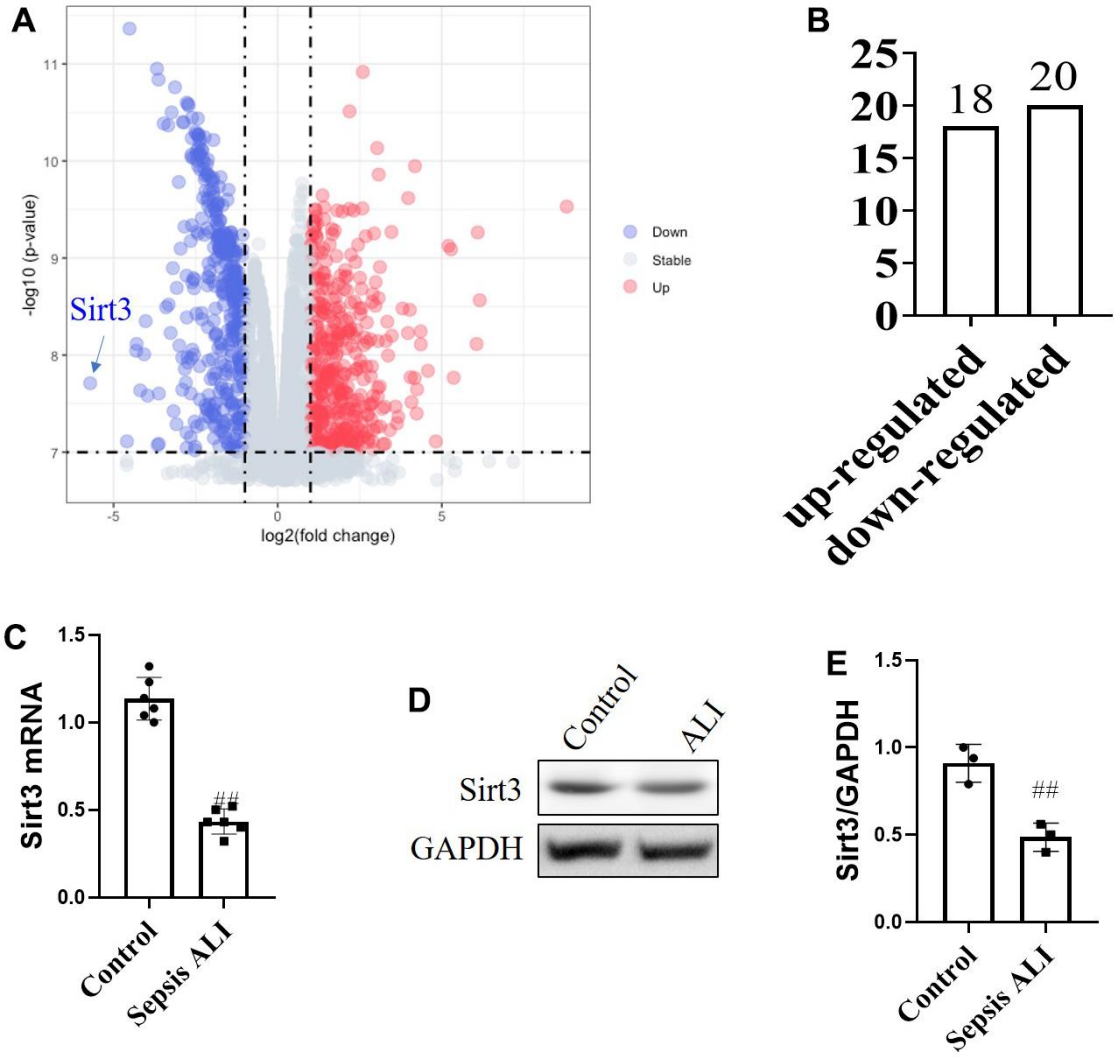
**Proteomic analysis of protein differences in sepsis with acute lung injury.** (**A**) Differential protein volcano map analysis; (**B**) Up and down regulation of protein; (**C**) mRNA levels of Sirt3; (**D**, **E**) protein levels of Sirt3. (*N*=6) All the data was presented as mean ± SD. Compared with control group: ^#^P<0.05, ^##^P<0.01.

The changes of lung tissue proteins in septic ALI mice and control mice were analyzed by proteomics. The results showed that 38 differential proteins were identified. Among them, 18 proteins were up-regulated and 20 proteins were down-regulated (Figure 1B). Through bioinformatics technology, it was found that Sirt3 was the protein with the most obvious changes, and it was significantly down-regulated (Figure 1A). In order to further verify the expression of Sirt3, we detected their mRNA and protein levels by PCR and Western blot. The results showed that compared with control mice, the mRNA level of Sirt3 was significantly decreased (Figure 1C) and the protein levels of Sirt3 was significantly decreased (Figure 1D, 1E).

### Effect and mechanism of PHL on ALI *in vivo* and *in vitro*

### In sepsis mice

**Figure 2:**
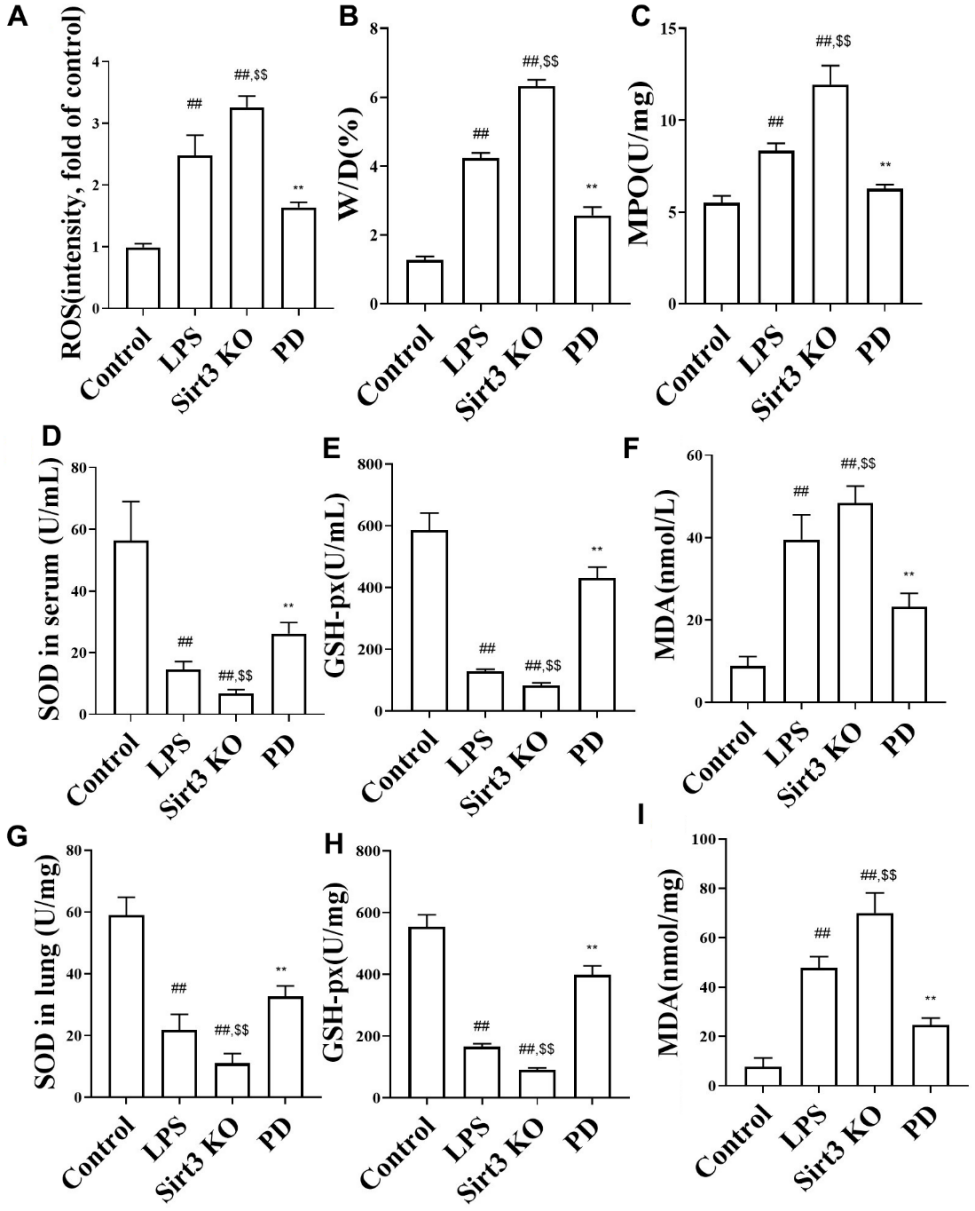
**The effects of PD on ROS, w/d, MPO and oxidative stress.** (**A**–**C**) ROS, w/d and MPO (n=6); (**D**–**F**) SOD, MDA and GSH-Px in serum (n=6); (**G**–**I**) SOD, MDA and GSH-Px in serum (n=6). All the data was presented as mean ± SD. Compared with control group: ^#^P<0.05, ^##^P<0.01. Compared with model group: ^*^P<0.05, ^**^P<0.01.

The effects of PD on the w/d, ROS and MPO in sepsis mice. As shown in Figure 2A–2C, the levels of w/d, ROS and MPO were markedly increased in model group than control group. Compare with LPS group, Sirt3 KO significantly increased levels of w/d, ROS and MPO. While, compared with LPS and Sirt3 KO groups, PD significantly decreased the levels of w/d, ROS and MPO.

The effects of PD on oxidative stress, inflammation and lung histopathology in sepsis mice. As shown in Figure 2D–2I, the levels of MDA in serum and lung tissues were increased, SOD and GSH-px in serum and lung tissues were decreased compared to the control group.

Further, Compare with LPS group, Sirt3 KO significantly decreased SOD and GSH-px in serum and lung tissues, increased MDA in serum and lung tissues. Compare with LPS group, Sirt3 KO significantly decreased the levels of SOD, GSH-px and increased MDA. While, compared with LPS and Sirt3 KO groups, PD significantly decreased MDA and increased SOD and GSH-px in serum and lung tissues.

**Figure 3:**
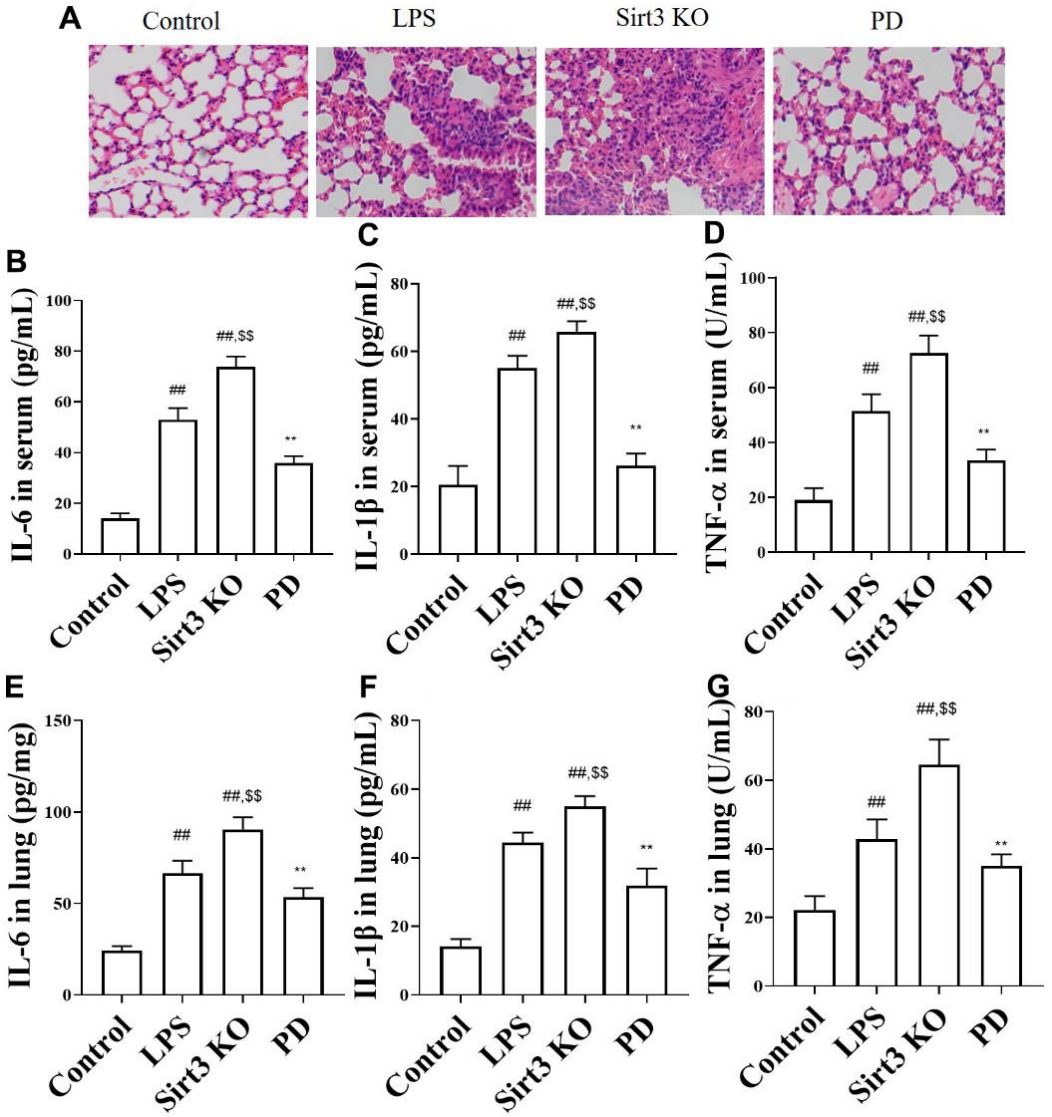
**The effects of PD on lung histopathology and cytokine in sepsis mice.** (**A**) Representative heart tissue sections photomicrographs for hematoxylin-eosin (HE) staining. Original magnification: 200, scale bar: 50 μm. (**B**–**D**) The levels of TNF-α, IL-1β, IL-6 in serum. (**E**–**G**) The levels of IL-6, TNF-a and IL-1β. All the data was presented as mean ± SD. Compared with control group: ^#^P<0.05, ^##^P<0.01. Compared with model group: ^*^P<0.05, ^**^P<0.01.

The histopathological test results of lung tissue after HE staining are shown in Figure 3A, which shown that the control group has regular lung tissue morphology, clear and complete alveolar structure, and no obvious bleeding or inflammatory cell infiltration, alveoli collapse, alveolar wall and alveolar septum thickening and inflammatory cell infiltration in lung tissue of ALI model group mice. Compare with LPS group, the above changes were more serious in Sirt3 KO group. While, compared with LPS and Sirt3 KO groups, PD significantly restored those changes.

In order to evaluate inflammatory reaction, cytokines (TNF-α, IL-1β and IL-6) were detected. As expected, the levels inflammatory cytokines TNF-α, IL-1β, IL-6 were increased in serum (Figure 3B–3D) and lung (Figure 3E–3G) in sepsis mice compared with control group. Compare with LPS group, Sirt3 KO significantly increased the levels of cytokines TNF-α, IL-1β, IL-6 in serum and lung, While, compared with LPS and Sirt3 KO groups, PD significantly decreased the levels of cytokines TNF-α, IL-1β, IL-6 in serum and lung.

**Figure 4:**
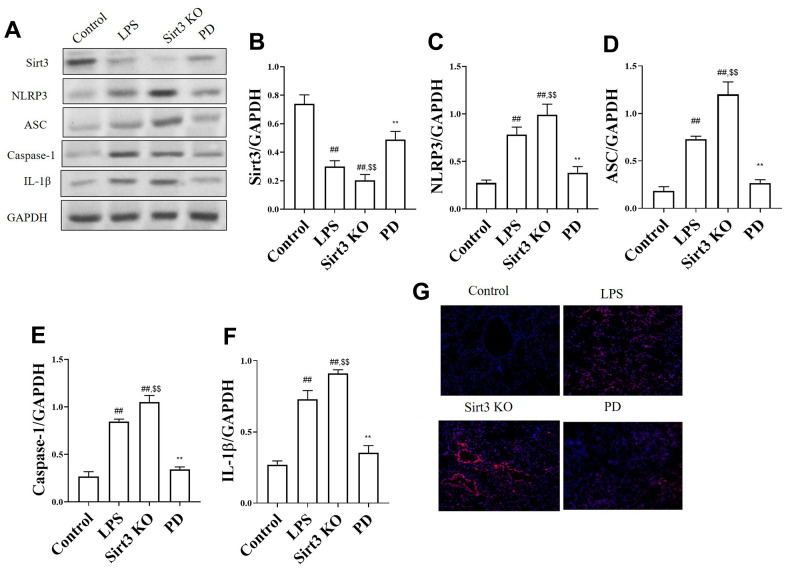
**The effects of PD on NOX4/ROS/NLRP3 pathway in sepsis mice.** (**A**–**F**) The levels of NLRP3, ASC, Caspase-1 and IL-1β; (**G**) Immunofluorescence of NLRP3 in lung (n=3). Original magnification: 200, scale bar: 20 μm. All the data was presented as mean ± SD. Compared with control group: ^#^P<0.05, ^##^P<0.01. Compared with model group: ^*^P<0.05, ^**^P<0.01.

The effects of PD on Sirt3/NLRP3 pathway associated protein in sepsis mice. As shown in Figure 4A–4F, compared with control group, the level of Sirt3 was significantly decreased, NLRP3, ASC, caspase-1 and IL-1β were increased. Compare with LPS group, Sirt3 KO significantly increased the levels of NLRP3, ASC, caspase-1, IL-1β. While, compared with LPS and Sirt3 KO groups, PD significantly increased the level of Sirt3, and decreased the levels of NLRP3, ASC, caspase-1 and IL-1β. The results of immunofluorescence were consistent with those of Western blot (Figure 4G).

### In MLE-12 cells

**Figure 5:**
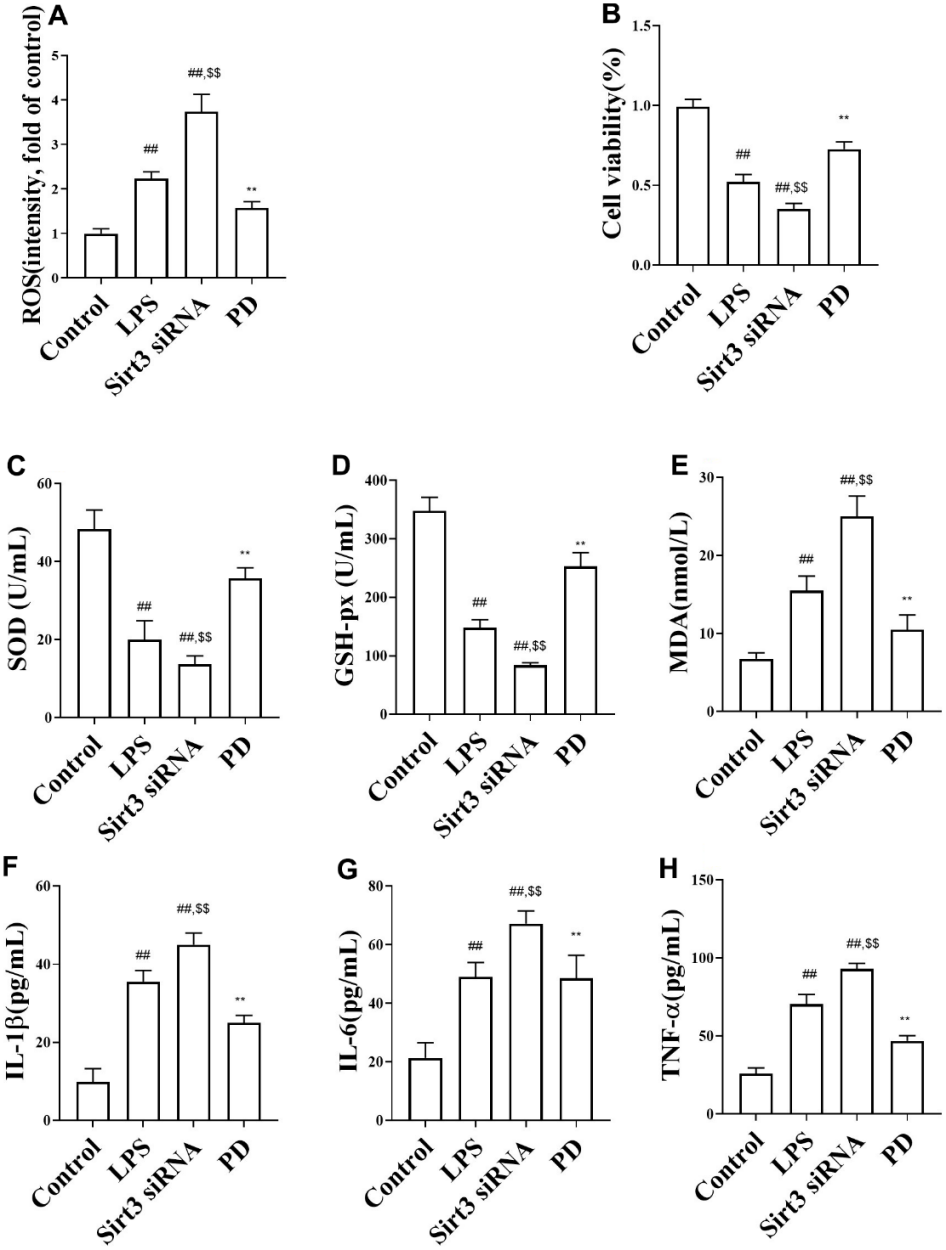
**The effects of PD on oxidative stress and cytokine LPS-induced MLE-12 cells.** (**A**, **B**) ROS and cell viability; (**C**–**E**) The level of SOD, MDA, GSH-Px; (**F**–**H**) The levels of TNF-a, IL-1β, IL-6. All the data was presented as mean ± SD. Compared with control group: ^#^P<0.05, ^##^P<0.01. Compared with model group: ^*^P<0.05, ^**^P<0.01.

The effects of PD on the cell viability and ROS in LPS-induced MLE-12 cells. As shown in Figure 5A, 5B, the level of cell viability was decreased and ROS were markedly increased in model group than control group. Compare with LPS group, Sirt3 KO significantly increased levels of ROS and decreased cell viability. While, compared with LPS and Sirt3 KO groups, PD significantly decreased the levels of ROS and increased cell viability.

The effects of PD on oxidative stress and inflammation in MLE-12 cells. As shown in Figure 5C–5E, the level of MDA in MLE-12 cells was increased, SOD and GSH-px in in MLE-12 cells were decreased compared to the control group. Further, Compare with LPS group, Sirt3 KO significantly decreased SOD and GSH-px in MLE-12 cells, increased MDA in in MLE-12 cells. While, compared with LPS and Sirt3 KO groups, PD significantly decreased MDA and increased SOD and GSH-px in MLE-12 cells.

As expected, the levels inflammatory cytokines TNF-α, IL-1β, IL-6 were increased in MLE-12 cells (Figure 5F–5H) compared with control group. Compare with LPS group, Sirt3 KO significantly increased the levels of cytokines TNF-α, IL-1β, IL-6 in MLE-12 cells, While, compared with LPS and Sirt3 KO groups, PD significantly decreased the levels of cytokines TNF-α, IL-1β, IL-6 in MLE-12 cells.

**Figure 6:**
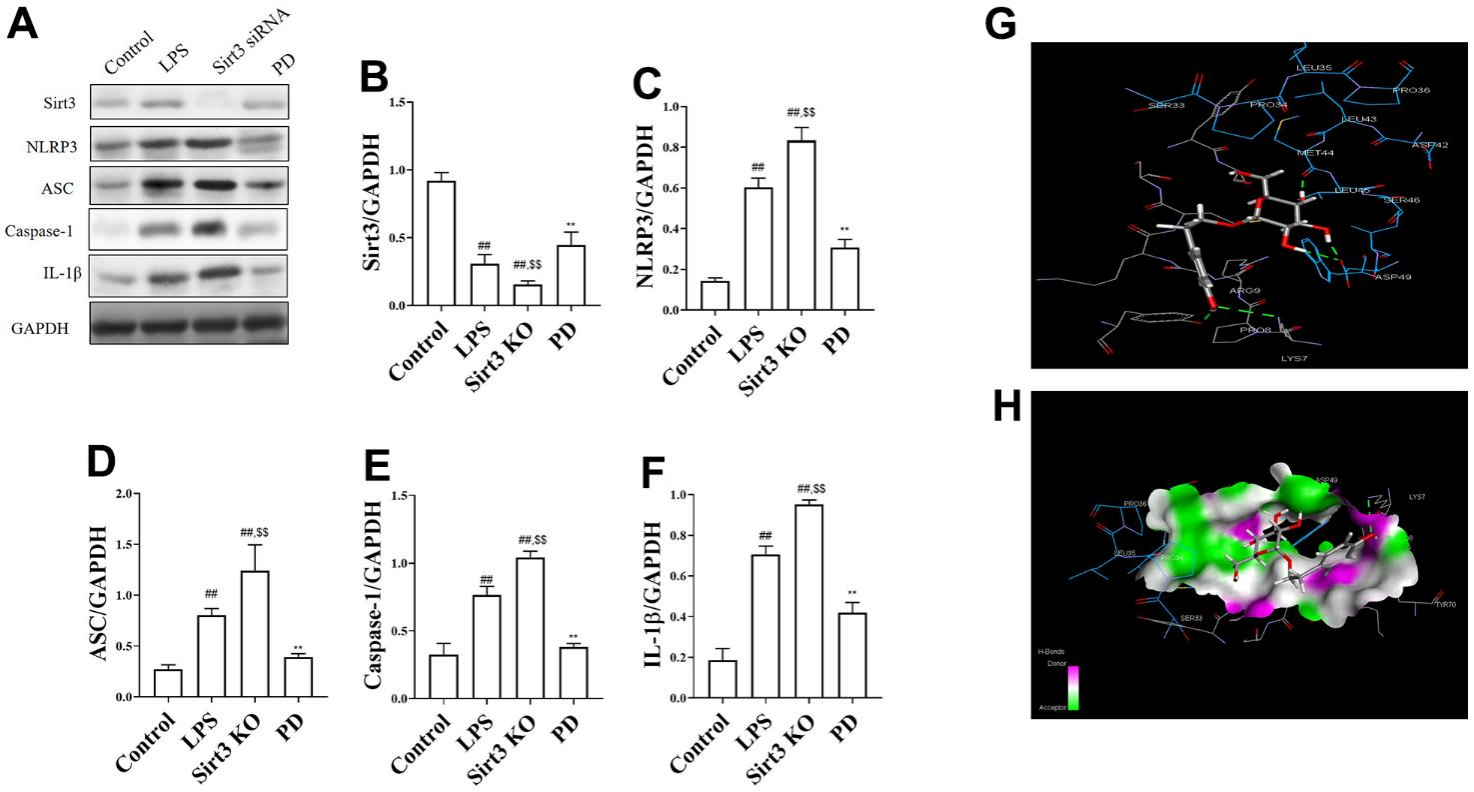
**The effects of PD on Sirt3/NLRP3 pathway in LPS-induced MLE-12 cells and molecules docking results of PD and Sirt3.** (**A**–**F**) The levels of NLRP3, ASC, Caspase-1 and IL-1β; (**G**, **H**) molecules docking results of PD and Sirt3. All the data was presented as mean ± SD. Compared with control group: ^#^P<0.05, ^##^P<0.01. Compared with model group: ^*^P<0.05, ^**^P<0.01.

The effects of PD on Sirt3/NLRP3 pathway associated protein in LPS induced MLE-12 cells. As shown in Figure 6A–6F, compared with control group, the level of Sirt3 was significantly decreased, NLRP3, ASC, caspase-1 and IL-1β were increased. Compare with LPS group, Sirt3 KO significantly increased the levels of NLRP3, ASC, caspase-1 and IL-1β. While, compared with LPS and Sirt3 KO groups, PD significantly increased the levels of Sirt3 and decreased the levels of NLRP3, ASC, caspase-1, IL-1β.

### Molecules docking results of PD and Sirt3

Molecules Docking technology is a convenient and effective means to explore the interaction between small molecules and target targets. Here, we used Vina 1.1.2 software to study the docking of compound PD with Sirt3 protein. As shown in the Figure 6G, 6H, the interaction diagram between the molecule PD and Sirt3 protein. The result that PD forms hydrogen bonds with arg-87, leu-67, arg-89, ile-70, asp-75, ser-88 and tyr-64 on Sirt3 protein. In addition, it also forms hydrophobic interaction with glu-65, ile-70, glu-65 and tyr-64, further strengthening the binding effect. A negative number of binding affinities indicates the possibility of binding. Generally, a value less than -6 kcal/mol is considered to be more likely to bind. In this complex, the binding affinity score given by docking software was -6.3 kcal/mol, which means that PD has potential active effect on Sirt3.

## DISCUSSION

ALI is an inflammatory response syndrome characterized by diffuse pulmonary edema, micro pulmonary atelectasis and progressive hypoxemia, which is caused by the injury of alveolar capillary [9, 10] endothelium and alveolar epithelium and the increase of microvascular permeability caused by various reasons. In severe cases, it can develop into acute respiratory distress syndrome (ARDS). Epidemiological research reports from many countries show that ALI seriously threatens the life and health of hospitalized patients [11]. At present, the clinical treatment of patients with ALI and ARDS mainly depends on mechanical ventilation, fluid management and other means, and there is a lack of more effective drug treatment. Therefore, it is particularly urgent to find more cellular pathways and mechanisms related to the occurrence and development of ALI, screen out accurately targeted signal pathways and sites, and find effective drugs to treat ALI. Through proteomic technology, this study found that there were 38 differentially expressed proteins in the lung tissues of sepsis mice and control mice, of which Sirt3 had the most significant difference, and the expression was significantly down-regulated. Further, we found that Plantainoside D (PD), an effective component of *Plantago asiatica L* could improve sepsis ALI by regulating Sirt3/NLRP3 pathway related proteins.

Oxidative stress plays an important role in septic lung injury. LPS induced excessive levels of reactive oxygen species (ROS), ROS accumulation triggers inflammation or cell death, which has been implicated in the pathogenesis of sepsis. Under physiological conditions, ROS production is counteracted by various cellular antioxidant enzymes to promote redox homeostasis [12, 13]. However, ROS accumulation could lead to an imbalance in favor of oxidants compared to antioxidant capacity. MDA, a marker of lipid peroxidation and antioxidant enzymes including SOD and GSH-Px, reflects the extent of oxidative stress. Our study showed that increased MDA and decreased SOD, GSH-Px in sepsis mice, indicated that increased ROS generation. PD treatment reduced ROS generation by decreased MDA and restored antioxidant enzymes including SOD and GSH-Px. At the same time, PD decreased ROS in mice and MLE-12 cells.

Inflammatory cytokines such as IL-1, IL-6, IL-18 and TNF-α are critically involved in the pathogenesis of sepsis [14]. IL-1β is synthesized by a variety of cell types, including macrophages, monocytes and fibroblasts, and is an effective mediator of inflammation and immunity. The sepsis animal models have been demonstrated that increase level of IL-1β [15]. IL-6 is another important inflammatory cytokine and IL-6 mRNA was demonstrated that in lung with sepsis and serum IL-6 is a candidate marker for sepsis [16]. TNF-α is a pro-inflammatory cytokine secreted from monocytes and macrophages that is functional in lipid metabolism, insulin resistance and endothelial biology. TNF-α involve in the pathogenesis of sepsis and inhibited it will have significant therapeutic effects [17]. PD markedly decreased the levels of cytokines in mice and MLE-12 cells showed that relieved sepsis-induced lung injury through inhibited inflammatory response.

Silent regulating type information regulation 2 homolog 3 (Sirt3) is a NAD dependent histone deacetylase. SIRT3 protein is widely expressed in tissues rich in mitochondria, such as kidney, heart, lung, brain and liver, which is involved in cell energy metabolism, proliferation, apoptosis and so on [18].

It has been reported that induction of Sirt3 expression could reduce inflammatory response [19, 20]. Next, we detected the interaction between Sirt3 and NLRP3 by Western blotting. The results showed that Sirt3 inhibited NLRP3. Some studies have shown that when tissues or cells are damaged, the concentration of extracellular ROS increases, which activates NLRP3, which further induces the maturation and release of IL-1β and initiates the inflammatory cascade reaction. Our study showed that PD markedly decreased the levels of NLRP3 both in mice and in MLE-12 cells.

In conclusion, Sirt3 is an important regulatory factor involved in acute lung injury in sepsis, we found that PD can significantly improve sepsis ALI, and its mechanism is related to the regulation of Sirt3/NLRP3 signal pathway, and the deeper mechanism needs to be further studied.

## MATERIALS AND METHODS

### Reagents

PD, specification: 20 mg, purity: > 98%, batch number: 2021-0576, produced by Sichuan Vicky biological company. Hematoxylin eosin (HE) staining solution was purchased from Solarbio Company, USA. Fetal blood serum, phosphate buffer (PBS) and DMEM medium were purchased from Hyclone Company, USA. Transfection reagent Transit-X2 was purchased from Shenzhen Minas Bio-Tech Co., Ltd., USA. Endotoxin-free plasmid small amount extraction kit was purchased from Qiagen Company of Germany. BCA protein quantitative kit was purchased from Beijing Pulilai Company, China and Western blot kit was purchased from Promega Company, USA. LPS was purchased from Sigma-Aldrich Company, USA. Diamino polyethylene glycol was purchased from Yarebio Company, China. All antibodies were purchased from Cell Signaling Technology Company, USA.

### Animals

WT mice (ICR, male, 6 weeks, 16-18g), Sirt3 KO mice (ICR, male, *Sirt3* ^-/-^, 6 weeks, 16-18g) were obtained from the Cyagen Biosciences Inc. All animals’ operations were approved by the Research Council and Animal Care and Use Committee of Animal Center of Suzhou University.

### Proteomics in ALI mice

Mice were divided into two groups, control mice and ALI mice. The ALI mice were administrated by intraperitoneal injection of lipopolysaccharide 10 mg/kg. The control group was given the same amount of saline in the same way. After 24 hours, the lung tissues of mice were collected for proteomic detection. Specific operation method according to previous reports [11].

### Establishment of ALI mouse model

ALI model of mice was established by intraperitoneal injection of lipopolysaccharide (LPS, 10 mg/kg). The WT mice (control group) was given the same amount of saline in the same way. Mice were divided into the following groups: WT mice (control), LPS, *Sirt3 KO* + LPS, *Sirt3 KO* + LPS + PD (50 mg/kg). After 24 hours of intervention, PD (50 mg/kg) (intraperitoneal injection) was given to mice by gavage for 7 consecutive days.

### *In vitro* model establishment

Lung epithelial cell line MLE-12 cells were seeded at 6-well-plate at a density of 1×10^6^ cells/well for 24 h. After that, the cells were divided into these groups: control, LPS (model, 50 μM), LPS + Sirt3 siRNA, LPS + Sirt3 siRNA + PD (5 μmol/L). The cells were treated with PD (5 μmol/L) for 24h, and 50 μM LPS were co-incubated for 24h, while the control group was challenged with an equal PBS. Cell supernatant and cells were collected for subsequent detection.

### Cell transfection

The MLE-12 cells were inoculated in a 6-well plate with 5×10^4^ cells per well, and cultured in DMEM containing 10% fetal bovine serum in an incubator based on 37° C and 5% CO2. When the cells grew to 50%-70% fusion degree, various shRNA were transfected with Translipid HL Transfection Reagent with a final concentration of 80 nmol/L according to the instructions of Translipid HL Transfection Reagent. Before transfection, the cells were cultured in serum-free DMEM medium 2 mL for 2 h, and after transfection for 6h, the conventional DMEM medium containing 10% fetal bovine serum was changed for further culture. Six hours after transfection, the expression of green fluorescent protein in MLE-12 cells was observed under fluorescence microscope. Ten low-power fields were randomly selected, and the transfection efficiency was evaluated by counting the percentage of transfected cells in the total number of cells. Western blot was used to evaluate the success of cell transfection. The small interfering RNA (siRNA) targeted for Sirt3 (5’-A C C C A G T G G C A T T C C A G A C3’, 5’-G G C T T G G G G T T G T G A A A G A A G-3’ and GAPDH, 5’-G C A C C G T C A A G G C T G A G A C-3’, 5’-T G G T G A A G A C G C C A G T G G A-3’ both synthesized by Sigma-Aldrich Company, USA, was designed on the basis of the Thomas Tuschl protocol. Lyophilized single-stranded RNA oligonucleotides were resuspended in sterile RNase free water (100 μM), denatured (heated at 95° C for 5 min), aligned and slowly annealed with decreasing temperature, resulting in the formation of double-stranded siRNA at 50 μM.

### Measurement of wet-dry ratio of lung (w/d)

The right lung of mice was removed. The trachea and esophagus were separated passively, the right lung lobe was obtained, and the wet weight was measured immediately. Subsequently, the lungs were dried at 60° C for 72 hours to remove all moisture, and then the dried lungs were weighed and the wet weight/dry weight ratio of the lungs was calculated.

### ROS and MPO test

10 mg of lung tissue was added with 1:10 normal saline, centrifuged at 12000r/min for 10min, and the supernatant was collected and detected by MPO and ROS commercial kit. The operation process strictly followed the instructions of the kits.

### Detection of oxidative stress

The levels of SOD, MDA and GSH-Px were detected by commercial kits, and the operation method was carried out according to the instructions.

### Cytokine measurement

The concentrations of IL-6, IL-1β and TNF-α in serum, lung tissues and cell supernatant were analyzed by enzyme-linked immunosorbent assay (ELISA) kits according to the manufacturer’s instructions.

### Hematoxylin-eosin (HE) staining

The lung was fixed in 4% formalin solution, embedded in paraffin, cut into 4μm thick sections, and placed on a glass slide for HE staining.

### Cell viability and ROS assay

MLE-12 cells used for cell viability, the medium was removed and 200 μL 0.5 mg/ml MTT was added to each well for 4 h. The supernatant was discarded and added 150 μl DMSO to each well. The absorbance value of each well was determined at 490 nm with a microplate spectrophotometer. For ROS assay, the detection was carried out according to the instructions of the kit.

### Immunohistochemistry

Lung tissue slices were baked at 60° C for 1 h, then paraffin was removed by xylene, dehydrated by gradient ethanol and heated by sodium citrate buffer for antigen repair. After natural cooling to room temperature, it was cultured with 3% hydrogen peroxide for 10 min. Each section was sealed with 3% BSA at room temperature. After removing the blocking solution, the section was incubated with the primary antibody at 4° C overnight, the secondary antibody was incubated for 10 min, and PBS was washed three times, each time for 3 min, the third antibody was incubated for 10 min and washed with PBS for 3 min each time. Samples were stained with DAB and hematoxylin, dehydrated by gradient ethanol and xylene, dried, sealed with neutral resin, and the expression of related proteins in lung tissue was observed under light microscope.

### Western blot analysis

Total protein was extracted from the lysate of lung tissue and MLE-12 cells, and the protein content was determined by BCA method. SDS-PAGE electrophoresis was performed on protein samples and transferred to PVDF membrane. The 5% skimmed milk powder was sealed at room temperature for 2 h, and incubated with related equal primary antibodies respectively. The membrane was rinsed with TBST and then reacted with horseradish peroxidase coupled secondary antibody. The membrane was rinsed with TBST and then developed with enhanced chemiluminescence (ECL) luminescent reagent. The optical density of the main band was measured by gray-scale imaging software (UVP, UK) to calculate the expression level of the above proteins in lung tissue.

### PD and Sirt3 molecular docking

The structures of Sirt3 and PD were drawn. After the energy of MM2 was minimized, it was further processed by Autodock tools1.5.6, and the torsion was set as the default value. Finally, it was saved as a pdbqt file for Autodock Vina to conduct molecular docking.

### Statistical analysis

All data were expressed as mean ± standards deviation (SD) and analyzed by one-way analysis of variance (ANOVA) followed by Tukey multiple comparison test using GraphPad prism 5 (GraphPad Software, USA). A value of *p<0.05* was considered statistically significant.

### Availability of data and material

Data can be requested by the corresponding author.
